# Validation of questionnaires to assess quality of life related to fecal incontinence in children with anorectal malformations and Hirschsprung's disease

**DOI:** 10.1016/j.rppede.2015.06.022

**Published:** 2016

**Authors:** Arthur Loguetti Mathias, Ana Cristina Aoun Tannuri, Mariana Aparecida Elisei Ferreira, Maria Mercês Santos, Uenis Tannuri

**Affiliations:** a Instituto da Criança do Hospital das Clínicas da Faculdade de Medicina da Universidade de São Paulo (USP), São Paulo, SP, Brazil; b Faculdade de Medicina da Universidade de São Paulo (USP), São Paulo, SP, Brazil

**Keywords:** Digestive system abnormalities, Hirschsprung's disease, Quality of life, Questionnaire, Children, Adolescents

## Abstract

**Objective:**

Surgical treatment of anorectal malformations (ARMs) and Hirschsprung's disease (HD) leads to alterations in bowel habits and fecal incontinence, with consequent quality of life impairment. The objectives were to create and validate a Questionnaire for the Fecal Incontinence Index (FII) based on the Holschneider score, as well as a Questionnaire for the Assessment of Quality of Life Related to Fecal Incontinence in Children and Adolescents (QQVCFCA), based on the Fecal Incontinence Quality of Life.

**Methods:**

The questionnaires were applied to 71 children submitted to surgical procedure, in two stages. Validity was tested by comparing the QQVCFCA and a generic quality of life questionnaire (SF-36), and between QQVCFCA and the FII. A group of 59 normal children was used as control.

**Results:**

At two stages, 45.0% (32/71) and 42.8% (21/49) of the patients had fecal incontinence. It was observed that the QQVCFCA showed a significant correlation with the SF-36 and FII (Pearson's correlation 0.57), showing that the quality of life is directly proportional to improvement in fecal incontinence. Quality of life in patients with fecal incontinence is still globally impaired, when compared with control subjects (*p*<0.05, Student's *t*-test). There were also significant differences between the results of children with ARMs and children with HD.

**Conclusions:**

QQVCFCA and FII are useful tools to assess the quality of life and fecal incontinence in these groups of children. Children with ARMs submitted to surgical procedure and HD have similar quality of life impairment.

## Introduction

Anorectal malformations and Hirschsprung's disease are congenital disorders affecting approximately 1:5000 live births. Surgical correction should be done early and the main objective is the anatomical reconstruction of structures with normal bowel habits.^1^ However, constipation and/or fecal incontinence are frequent, with important consequences on personal, social, and professional spheres that may reflect in adulthood. Thus, patients may suffer strong negative impact on quality of life (QoL). QoL is defined by the World Health Organization as “the individual's perception of their position in life in the context of the culture and value systems in which they live and in relation to their goals, expectations, standards and concerns”.^2^ Thus, QoL is a subjective data comprising several areas and should be evaluated individually and based on the expectations of patients and their relatives.

In the Pediatric Surgery and Liver Transplantation Service of the Children's Institute of Hospital das Clínicas da Faculdade de Medicina da USP—where 15 new cases are attended on average per year—fecal continence is assessed subjectively on three levels (good, fair and poor) that do not objectively reflect reality. In this service, the issue of quality of life is not thorough. Only cases in which psychosocial problems are externalized or are more serious are referred to a psychologist or social worker. Thus, it became necessary to apply questionnaires to evaluate physical (fecal continence) and psychosocial (quality of life) performance of patients, so that pediatrician and surgeon can perform effective and positive interventions during the follow-up. We did not find instruments that met the needs of our target population in the literature. The objective of this study was to develop and validate new questionnaires to assess quality of life and fecal incontinence, from a population of children undergoing surgery for anorectal malformation repair or Hirschsprung's disease in clinical follow-up in our clinic.

## Method

The Questionnaire for the Fecal Incontinence Index (FII), based on the Clinical Evaluation of Fecal Continence (Holschneider Criteria)^3^ and the Questionnaire for the Assessment of Quality of Life Related to Fecal Continence in Children and Adolescents (QQVCFCA), based on the Fecal Incontinence Quality of Life (FIQL) were created.^4^ The created questionnaires were submitted to the translation and cultural adaptation processes, then the validation step was initiated. For this, they were sent along with the Short-Form 36 (SF-36)^5^ questionnaire via mail to volunteers, and without the presence of an interviewer.

The Clinical Evaluation of Fecal Continence (Holschneider Criteria) is an established and widely used index in pediatric surgery; however, it has not validated translation into Portuguese and its questions are fit for interview application. Thus, the Fecal Incontinence Index (FII; available from corresponding author) was created based on Holschneider Criteria, consisting of 8 questions easily understood on procedures of everyday life (questions 1–5) and on diarrhea, constipation, and use of auxiliary treatment (questions 6–8). It meets the Holschneider's criteria. Thus, it was possible to create a scoring system of 0–6, with value of 0–2 points for each question. The range of 0–5 indicates poor continence, 6–10 fair continence, 11–15 good continence and maximum score, and 16 normal fecal continence.

The questionnaire to assess quality of life related to fecal continence in children and adolescents (QQVCFCA; available from corresponding author) was based on the translation into Portuguese and validation of FIQL. FIQL is a questionnaire of 29 questions for use in adults, which has questions that are considered repetitive and some that address situations of severe depression and sex, considered unsuitable for children and adolescents. Thus, 16 questions were not used; the wording of 10 questions was modified but kept the same meaning; 3 questions were maintained; and 5 were added, totaling 18 questions. The fifth option, “none of the answers”, was changed to “other cause”, which proved to be more in line with the explanatory text. This standard questionnaire was submitted to analysis by a multidisciplinary team that changed it: the QQVCFCA was expanded from 18 to 24 questions; and the fifth option, “other cause” has been deleted. At that time, the suggestion was to leave the answer blank. The options “I strongly agree”, “I somewhat agree”, “I disagree a bit”, and “I strongly disagree” were considered difficult to understand and replaced by “almost always”, “some times”, “rarely”, and “never”. The same domains of the original were covered: lifestyle (7 questions), behavior (7 questions), depression (7 questions), and embarrassment (3 issues). Questions 22 and 23 are related to patient's opinion and satisfaction with their own health and bowel function. Each question has a score of 1–4 (1=worst situation). The final score is obtained by summing the mean score obtained in each domain, and ranges from 4 to 16.

The SF-36 (Medical Outcomes Study 36-Item Short-Form Health Survey) is a generic tool for assessing the quality of life, which is easy to administer and understand. It is a multidimensional questionnaire consisting of 36 items, grouped in eight domains: physical functioning, bodily pain, general health, vitality, social functioning, emotional aspects, and mental health. This questionnaire was also applied and the results were compared with the results obtained in QQVCFCA.

The questionnaires were administered in 85 patients, between 4 and 19 years, undergoing anorectal repair for malformations or Hirschsprung's disease, with follow-up in the clinic. Only 10 patients had 4–6 years, value considered insufficient. Therefore, they were not used in this study. Of the 75 remaining patients, 4 responded only to the FII and were also excluded. Thus, 71 patients, from 7 to 19 years, who had anorectal malformations or Hirschsprung's disease, whose surgical treatment has been completed for at least six months, and who agreed to participate were enrolled. Patients with some degree of impaired neuropsychomotor development and neurological and urinary disorders involving the sphincter control were excluded. Of the 71 cases, 31 were Hirschsprung's disease: 23 cases of classic form (rectosigmoid aganglionosis); 7 cases of total colic aganglionosis; one case of intestinal neuronal dysplasia. Of the total, 22 were male and 9 female. The remaining cases, 40 were of anorectal malformations: 8 low form; 15 intermediate and/or high form; 4 persistent cloaca; one vesicular-intestinal fissure; one rectal atresia; 11 not reported (children operated in other services referred to the Institution due to intestinal constipation).

Control group consisted of 59 healthy individuals without bowel function complaints and demographically similar to the assessed patients. They were selected in outpatient clinic of pediatric surgery and suffered from common surgical conditions, such as inguinal hernia and phimosis. Similar to the study groups, the questionnaires were sent by mail.

The measurement properties studied were reproducibility and validity. To evaluate reproducibility, FII and QQVCFCA were sent twice to the volunteers by mail. Between the first and second sending, there was an interval of 6 days to 14 months. Of the 71 initial patients, 22 did not respond to the second sent questionnaires. Of the 49 remaining patients, 24 have anorectal malformations and 25 have Hirschsprung's disease.

Convergent and discriminant validity was tested. To assess the construct validity, incontinent patients also responded to the generic questionnaire known as Short Form-36, already validated in our midst. The results were correlated with those of QQVCFCA and then the FII and QQVCFCA results were compared. Results of patients with anorectal malformations and Hirschsprung's disease were also compared. Discriminant validity was evaluated with the application of the questionnaires in 71 operated patients and 59 healthy volunteers. The mean scores for each group on FII, QQVCFCA, and each of its domains were compared.Bray–Curtis index, Pearson's coefficient, and Student's *t*-test were used. The significance level was 0.05.

## Results

Based on the FII, it was found that among the initial 71 patients, continence was poor in 16, fair in 16, good in 26, and normal in 13 patients. Among 49 patients who also participated in the second phase, it was found that of the 25 with Hirschsprung's disease, 6 had poor, 6 had fair, 9 had good, and 4 had normal continence. Among the 24 with anorectal malformations, continence was poor in 4, fair in 5, good in 12, and normal in 3 patients. In short, 45.07% (32/71) and 42.85% (21/49) of patients had fecal incontinence in the two phases of questionnaire application.

The reproducibility of the results obtained from both questionnaires could be seen over time through their application in the same patients at two different times. The interval between these times ranged from 6 days to 14 months. The first application was termed FII1 and QQVCFCA1 and the second FII2 and QQVCFCA2.

The similarity between the first and second questionnaires proved to be satisfactory according to the Bray–Curtis index (>0.80). The reproducibility over time remained in all evaluated domains [FII, QQVCFCA, lifestyle (LS), behavior (BEH), depression (DEP), and embarrassment (EMB)], regardless of the time interval between questionnaire applications (Table 1).

**Table 1:** Comparison of similarity between FII 1 and 2 and QQVCFCA 1 and 2 and its domains.

Time interval between questionnaire applications	FII1×FII2	QQVCFCA1×QQVCFCA2	LS1×LS2	BEH1×BEH2	DEP1×DEP2	EMB1×EMB2
6 days to 1 month	0.95	0.96	0.94	0.97	0.93	0.92
2 to 3 months	0.92	0.93	0.89	0.96	0.92	0.90
4 to 6 months	0.92	0.96	0.94	0.97	0.95	0.94
6 to 14 months	0.96	0.94	0.95	0.93	0.94	0.85
All	0.94	0.95	0.95	0.96	0.93	0.90

FII, fecal incontinence index; QQVCFCA, questionnaire for the assessment of quality of life related to fecal incontinence in children and adolescents; LS, lifestyle; BEH, behavior; DEP, depression; and EMB, embarrassment. The number 1 after the criterion relates to the first application of the questionnaires; the number 2 refers to the second application of the same questionnaires. *Note* : Bray–Curtis Index: 0–0.19 – not similar; 0.20–0.39 – little similarity; 0.40–0.59 – medium similarity; 0.60–0.79 – high similarity; 0.80–1.0 – maximum similarity.

To assess construct validity, Pearson correlation was used and values from 0.50 to 0.74 were considered as reasonable correlation and values between 0.75 and 0.99 as strong correlation. Direct correlation was observed with the value of 0.5 between the results of QQVCFCA and SF-36, applied to 71 patients. Thus, the results for quality of life are similar with the application of the SF-36 or QQVCFCA. Comparing FII and QQVCFCA, it is noted that there is a dependency between them (index of 0.82). There is also a strong correlation between the FII with each domain of QQVCFCA individually (lifestyle: 0.75; behavior: 0.78; absence of depression: 0.78; absence of embarrassment: 0.71). Thus, the better the results of fecal incontinence, the better the quality of life and all parameters evaluated in the domains.

As for discriminant validity, it was found that the quality of life of fecal incontinent patients is still hampered globally in all domains and FII (mean scores of quality of life: 11.2; lifestyle: 3.1; behavior: 3.0; depression: 2.6; embarrassment: 2.3, and FII: 10.4) compared with healthy volunteers (15.2; 3.9; 3.9; 3.6; 3.9, and 15.4, respectively), and there is a significant statistical difference between groups when Student's *t*-test was applied, with *p*<0.05 (Table 2). Moreover, there was no significant difference between the results of children with anorectal malformations and Hirschsprung's disease (Table 3).

**Table 2:** Comparison of FII and QQVCFCA and its domains between healthy volunteers (group “normal”) and children with anorectal malformations or Hirschsprung's disease (group “patient”).

		Mean	Standard deviation	25th percentile	75th percentile	Median
*ICF*						
	Normal	15.37	1.0	15.0	16.0	16.0
	Patient	10.4	4.48	6.0	15.0	11.0
*QQVCFCA*						
	Normal	15.2	0.9	15.0	15.7	15.5
	Patient	11.2	3.3	8.6	14.8	11.2
*LS*						
	Normal	3.9	0.3	4.0	4.0	4.0
	Patient	3.1	0.9	2.3	4.0	3.4
*BEH*						
	Normal	3.9	0.2	3.8	4.0	4.0
	Patient	3.0	0.8	2.5	3.8	3.1
*DEP*						
	Normal	3.6	0.31	3.4	3.8	3.6
	Patient	2.7	0.83	2.1	3.5	2.7
*EMB*						
	Normal	3.9	0.2	4.0	4.0	4.0
	Patient	2.3	1.11	1.3	3.6	2.0

FII, fecal incontinence index; QQVCFCA, questionnaire for the assessment of quality of life related to fecal incontinence in children and adolescents; LS, lifestyle; BEH, behavior; DEP, depression; and EMB, embarrassment. Performed with Student's *t* -test, with *p* <0.05 and statistically different results.

**Table 3:** Comparison of FII and QQVCFCA and its domains between children with anorectal malformations and Hirschsprung's disease.

		Mean	Standard deviation	25th percentile	75th percentile	Median
*ICF*						
	HD	10.0	4.4	5.5	13.5	11.0
	ARM	10.9	4.6	6.5	15.0	13.0
*QQVCFCA*						
	HD	11.2	3.4	8.1	15.0	11.0
	ARM	10.8	3.6	7.6	14.9	10.5
*LS*						
	HD	3.1	0.8	2.3	4.0	3.5
	ARM	3.1	0.8	2.3	4.0	3.0
*BEH*						
	HD	3.1	0.8	2.5	4.0	3.3
	ARM	2.9	0.9	2.2	3.7	3.2
*DEP*						
	HD	2.7	0.8	1.8	3.4	2.5
	ARM	2.5	1.0	1.6	3.8	2.3
*EMB*						
	HD	2.2	1.2	1.2	3.6	1.6
	ARM	2.3	1.1	1.0	3.4	2.0

HD, Hirschsprung's disease; ARM, anorectal malformations; FII, fecal incontinence index; QQVCFCA, questionnaire for the assessment of quality of life related to fecal incontinence in children and adolescents; LS, lifestyle; BEH, behavior; DEP, depression; and EMB, embarrassment.

## Discussion

About 30–50% of patients with anorectal malformations or Hirschsprung's disease may have some degree of fecal incontinence,^6^ similarly to what was observed in this study. In general, it affects the occupational, social, emotional, sporting, and sexual areas and may lead to psychiatric disorders with loss of individual independence. It is therefore closely related to QoL.^7^ In a review article of several studies, it was shown that patients with impaired bowel function also have impaired QoL, with this correlation ranging from small, medium or strong.^1^ Despite advances in surgical pediatric techniques, fecal incontinence remains a common problem for patients.^8^ Therefore, during outpatient care, just asking about the number of bowel movements and subjectively evaluating the patient status has proven insufficient and superficial. It is important to include the concept of QoL; that is, the extent to which these physical/psychological/social problems affect the functionality and expectations of the patient. Therefore, the assessment of QoL and fecal incontinence should be done using objective, quantitative, and reproducible methods that allow long-term follow-up of patients.

There are several tools for that purpose in the literature that are specific and validated, such as the Fecal Incontinence Questionnaire,^9^ Fecal Incontinence Severity Index,^10^ Fecal Incontinence Quality of Life (FIQL),^11^ Hirschsprung's Disease Anorectal Malformation QoL Questionnaire (HAQL),^12^ Medical Outcomes Study 36-Item Short-Form Health Survey,^13^ and Clinical Evaluation of Continence (Holschneider Criteria). In the present investigation, the questionnaires used were translated and adapted into Portuguese. All cases were evaluated by the main investigator (ALM), the participation of the original authors was considered infeasible for practical reasons. Such conduct was also supported by the Institutional Review Board of the Institution.

The HAQL, for example, is specifically designed for patients with anorectal malformations or Hirschsprung's disease. It is a survey with 39–42 questions answered by the patient or their parents/guardians. It consists of 10–11 subscales, with items related to laxative diet, constipating diet, diarrhea, fecal incontinence, urinary continence, social functioning, emotional functioning, body image, physical symptoms, and sexual functioning. The answers are provided by the patient, by the parents, or both, depending on age. Thus, there is a form for parents of children aged 6 and 7 years; a form for parents with children 8–11 years and adolescents 12–16 years; and an adult form for patients aged 17 years or more.^13^

The FIQL, in turn, validated in English in 2000, was designed to evaluate the quality of life in any patients with fecal incontinence. It consists of 29 items assessing four domains: lifestyle, behavior, depression, and embarrassment. The same questionnaire was used, regardless of patient age.^10^

Although many instruments are found in the literature, none is adequate to address the target population of Instituto da Criança, São Paulo, Brazil. Part of the population attended at the Institute has low cultural level, making it difficult/impossible to apply questionnaires in other languages. There are also questionnaires with options considered complex and difficult to understand, such as the FIQL that has the following options: “strongly agree”, “somewhat agree”, “somewhat disagree”, “strongly disagree”, and “not applicable”. In addition, some patients fail to regularly attend the clinic about 2–3 years after the surgical treatment due to poor adherence to treatment or even to difficult access to health care. Still, many patients live in remote areas and have few resources for transportation, which complicates the application and interviews. Finally, we also found questions considered inappropriate for children involving sexuality and situations of severe depression. Thus, this study was necessary and describes the translation, cultural adaptation, and validation of two questionnaires, FII and QQVCFCA, congruent with ARMs or HD patients. FIQL and Holschneider Criteria, presented above, were used as models.

The process of translation, cultural adaptation and validation of questionnaires has been made in other countries. Minguez et al. validated the FIQL into Spanish, kept the 29 questions and applied it in patients with mean age of 60 years with fecal incontinence of any etiology. Incontinence severity was also rated and its relation to domains of FIQL.^12^ Rullier et al. validated the FIQL into French, with a study similar to that by Minguez et al., and 100 patients with mean age of 57 years were evaluated.^7^ In Sweden, Wigander et al. translated and culturally adapted the HAQL. The questionnaires had two extra questions regarding the original HAQL: the first asked if the patient had difficulty understanding the questions or if they found it odd; the second, if there was something they would like to add.^13^ In Brazil, Yusuf et al. validated the FIQL into Portuguese, but without adapting it to children and adolescents, the mean age of patients was 52.8 years.^14^

In all these studies, the questionnaires and assessment tools of QoL and fecal continence underwent a detailed process of translation and cultural adaptation, in order to suit the beliefs, customs, daily life, behavior, and socio-economic conditions of the target population. In the present study, the importance of this step was perceived by the significant number of fundamental changes in the original questionnaires used, such as suppression of questions, change in the wording of questions, creating questions and changing options. In addition, a multidisciplinary team was necessary to structure and, after an initial assessment, modify once more the questionnaires again. This thorough process ensures an improvement of the final questionnaires.

Moreover, reproducibility and validity of instruments were tested to evaluate the reliability. Reproducibility proved stable, with indices always above 0.85. Thus, it is concluded that there was a significant similarity in the results of the questionnaires in two separate applications in the same patients. The time interval between applications ranged from 6 days to 14 months, with no change in the results as well. The proven reproducibility in the time interval of 6 days to 1 month is especially important. In a short period, sudden changes in the evolution of all patients would be unlikely, which could compromise the results. In a longer period, the reproducibility could not have been proven due to changes in the clinical status of patients, if interventions with more pronounced effects had been made.

During the validation phase, there was a reasonable correlation between SF-36 and QQVCFCA, with a coefficient of 0.57 considered statistically significant. It confirms that the analyzed questionnaire has adequate validity when compared with a sensitivity instrument already established. It was also seen a direct relationship between the FII and QQVCFCA. This showed that quality of life is affected in patients with poor control of fecal continence. This relationship was also seen between FII and QQVCFCA's domains; so that, the better the fecal continence, the better the lifestyle and behavior indices and absence of depression and embarrassment. Finally, in the discriminative validation, it was found that in patients with anorectal malformations or Hirschsprung's disease the scores are lower than in the control population, which proves the impact of fecal incontinence on quality of life of patients. It was found that the greatest impact on quality of life of patients occurs in the domains of depression and embarrassment.

The loss to follow-up of 22 (30%) patients during the reproducibility process over time may be regarded as a study limitation. These patients may not have understood that it was a research process in which the questionnaire was sent twice intentionally and not accidentally. Thus, improving the understanding of volunteers is needed during research activities.

The association, in particular, of anorectal malformations with other congenital and genetic diseases is common and affects a significant percentage of patients attended at ICr. Thus, patients with Down syndrome, for example, were not included in the inclusion criteria due to the presence of other factors that could interfere with the evaluation of quality of life, confounding factors. Thus, there is a great need for the creation and validation of specific methods to assess such children, in terms of both fecal continence and quality of life.

Finally, it is also important that young children who were not included in this study because the number (only 10) was considered unrepresentative of the total be evaluated in the future. In this case, different questionnaires and forms of recreational evaluation could be proposed, due to difficulty of understanding and abstraction necessary for the answers.

In conclusion, the creation of the FII and QQVCFCA followed the trend of several centers around the world to create and validate their own questionnaires. The validation process was satisfactory and provided instruments in Portuguese for evaluation of children with anorectal malformations and Hirschsprung's disease. It can be used both in the medical practice and in surveys. It is also a model that standardizes the evaluation, allowing its use in other centers.
