# Supplementary material for: Validation of questionnaires to assess quality of life related to fecal incontinence in children with anorectal malformations and Hirschsprung's disease
Source: Rev Paul Pediatr. 2016 Jan-Mar;34(1):99–105. doi: 10.1016/j.rppede.2015.06.022 (PMC4795728; doi:10.1016/j.rppede.2015.06.022)
Supplement: Translation

# Translation: Validação de questionários para avaliação da qualidade de vida relacionada à continência fecal em crianças com malformações anorretais e doença de Hirschsprung

**Authors:** Arthur Loguetti Mathias, Ana Cristina Aoun Tannuri, Mariana Aparecida Elisei Ferreira, Maria Mercês Santos, Uenis Tannuri

**Affiliations:** a Instituto da Criança do Hospital das Clínicas da Faculdade de Medicina da Universidade de São Paulo (USP), São Paulo, SP, Brasil; b Faculdade de Medicina da Universidade de São Paulo (USP), São Paulo, SP, Brasil

**Keywords:** Anomalias do sistema digestivo, Doença de Hirschsprung, Qualidade de vida, Questionário, Crianças, Adolescentes

## Abstract

**Objetivo:**

O tratamento cirúrgico das malformações anorretais (MAR) e da doença de Hirschsprung (DH) leva a alterações do hábito intestinal e incontinência fecal com prejuízo da qualidade de vida. Os objetivos foram criar e validar o Questionário para o Índice de Continência Fecal (ICF), baseado no Holschneider Criteria, bem como o Questionário para Avaliar a Qualidade de Vida Relativa à Continência Fecal em Crianças e Adolescentes (QQVCFCA), baseado no Fecal Incontinence Quality of Life.

**Métodos:**

Os questionários foram aplicados em 71 crianças operadas, em duas etapas. A validade foi testada por meio da comparação do QQVCFCA e um questionário genérico de qualidade de vida (SF-36) e entre o QQVCFCA e o ICF. Um grupo de 59 crianças normais foi usado como controle.

**Resultados:**

Nas duas etapas, 45,0% (32/71) e 42,8% (21/49) dos pacientes apresentaram incontinência fecal. Verificou-se que o QQVCFCA apresentou correlação significativa com o SF-36 e o ICF (correlação de Pearson 0,57) e mostrou que a qualidade de vida é diretamente proporcional à melhoria da continência fecal. A qualidade de vida no paciente com incontinência fecal está ainda comprometida globalmente, em comparação com os indivíduos controles (*p*<0,05; teste *t* de Student). Não houve ainda diferença significativa entre os resultados de crianças com MAR e crianças com DH.

**Conclusões:**

O QQVCFCA e o ICF são instrumentos úteis para a avaliação da qualidade de vida e da incontinência fecal nesses grupos de crianças. Crianças operadas de MAR e DH apresentam comprometimentos semelhantes da qualidade de vida.

## Introdução

As malformações anorretais e a doença de Hirschsprung são afecções congênitas com incidência aproximada de um em cada 5.000 nascidos vivos. A correção cirúrgica deve ser feita precocemente e o principal objetivo é a reconstrução anatômica das estruturas com hábitos intestinais normais.^1^ No entanto, obstipação e/ou incontinência fecal são frequentes, com consequências importantes nos âmbitos pessoal, social e profissional que podem repercutir até na vida adulta. Assim, os pacientes podem sofrer forte impacto negativo na qualidade de vida (QdV). Essa é definida pela Organização Mundial da Saúde como “a percepção do indivíduo de sua posição na vida no contexto da cultura e sistema de valores nos quais ele vive e em relação aos seus objetivos, expectativas, padrões e preocupações”.^2^ Assim, QdV é um dado subjetivo, que abrange diversas áreas e deve ser avaliado individualmente e baseado nas expectativas do paciente e de seus parentes.

No Serviço de Cirurgia Pediátrica e Transplante Hepático do Instituto da Criança do Hospital das Clínicas da Faculdade de Medicina da USP, onde são atendidos, em média, 15 novos casos por ano, a continência fecal é avaliada de modo subjetivo, em três níveis (bom, regular e ruim) que não traduzem objetivamente a realidade. Nesse atendimento, a questão da qualidade de vida não é aprofundada. Apenas os casos em que os problemas psicossociais se exteriorizam ou são mais graves são encaminhados ao psicólogo ou ao serviço social. Logo, tornou-se necessária a aplicação de questionários que avaliem o desempenho físico (continência fecal) e psicossocial (qualidade de vida) dos pacientes, de modo que pediatra e cirurgião possam fazer intervenções efetivas e positivas ao longo do seguimento ambulatorial. Na literatura, não foram encontrados instrumentos que atendessem às necessidades de nossa população-alvo. Os objetivos da presente investigação foram os de criar e validar novos questionários para avaliação da qualidade de vida e incontinência fecal, a partir de uma população de crianças operadas para correção de malformação anorretal ou doença de Hirschsprung, em seguimento clínico em nosso ambulatório.

## Método

Foram criados o Questionário para o Índice de Continência Fecal (ICF), baseado no Clinical Evaluation of Faecal Continence (Holschneider Criteria),^3^ e o Questionário para Avaliar a Qualidade de Vida Relativa à Continência Fecal em Crianças e Adolescentes (QQVCFCA), baseado no Fecal Incontinence Quality of Life (FIQL).^4^ Os questionários criados foram submetidos aos processos de tradução e adaptação cultural e, em seguida, foi iniciada a etapa de validação. Para tanto, eles foram enviados, juntamente com o Questionário Short-Form 36 (SF-36)^5^ via correio, para os voluntários, portanto sem a presença de um entrevistador.

O Clinical Evaluation of Faecal Continence (Holschneider Criteria) é índice consagrado e muito usado em cirurgia pediátrica, entretanto não tem tradução validada para a língua portuguesa e suas questões são próprias para aplicação em entrevista. Assim, criou-se o Índice de Continência Fecal (ICF; disponível com autor correspondente), baseado no Holschneider Criteria, composto de oito questões de fácil compreensão, sobre procedimentos do dia a dia (questões 1 a 5) e sobre diarreia, constipação e uso de tratamentos auxiliares (questões 6 a 8). Atende aos quesitos de Holschneider. Dessa forma, foi possível criar um sistema de pontuação de 0 a 16, com valor de 0 a 2 pontos para cada questão. A faixa de 0 a 5 indica má continência, a de 6 a 10 continência regular, a de 11 a 15 continência boa e a pontuação máxima, 16, continência fecal normal.

O questionário para avaliar a qualidade de vida relativa à continência fecal em crianças e adolescentes (QQVCFCA; disponível com o autor correspondente) baseou-se na tradução para a língua portuguesa e validação do FIQL. O FIQL é questionário de 29 perguntas, para uso em adultos, que apresenta questões consideradas repetitivas e algumas que abordam situações de grave depressão e sexo, consideradas inadequadas para crianças e adolescentes. Assim, 16 questões não foram usadas; 10 tiveram redação modificada, mas com o mesmo sentido; três foram mantidas; e cinco acrescentadas, total de 18 questões. A quinta opção, “nenhuma das respostas”, foi mudada para “outra causa”, que se provou mais de acordo com o texto explicativo. Esse questionário modelo foi submetido à análise de uma equipe multiprofissional, que o modificou: o QQVCFCA foi ampliado de 18 para 24 questões; e a quinta opção, “outra causa”, foi suprimida. Sugeriu-se, naquelas circunstâncias, deixar a resposta em branco. As opções “concordo fortemente”, “concordo um pouco”, “discordo um pouco”, “discordo fortemente” foram consideradas de difícil compreensão e substituídas por “quase sempre”, “algumas vezes”, “raramente” e “nunca”. Foram compreendidos os mesmos domínios do original: estilo de vida (sete questões), comportamento (sete questões), depressão (sete questões) e constrangimento (três questões). As questões 22 e 23 estão relacionadas com a opinião e satisfação do paciente com a própria saúde e com o funcionamento do intestino. Cada questão tem uma nota de 1 a 4, 1 é a pior situação. A nota final é obtida por meio da soma da pontuação média obtida em cada domínio e varia de 4 a 16.

O SF-36 (Medical Outcomes Study 36–Item Short–Form Health Survey) é um instrumento genérico de avaliação da qualidade de vida, de fácil administração e compreensão. Trata-se de um questionário multidimensional formado por 36 itens, englobados em oito domínios, que são: capacidade funcional, aspectos físicos, dor, estado geral da saúde, vitalidade, aspectos sociais, aspectos emocionais e saúde mental. Esse questionário também foi aplicado e seu resultado foi comparado com o resultado obtido no QQVCFCA.

Os questionários foram aplicados em 85 pacientes entre quatro a 19 anos, operados para correção de malformação anorretal ou doença de Hirschsprung, em seguimento no ambulatório. Apenas dez pacientes tinham de quatro a seis anos, valor considerado insuficiente. Logo, não foram usados neste estudo. Dos 75 pacientes restantes, quatro responderam apenas ao ICF e também foram eliminados. Assim, foram estudados 71 pacientes, de sete a 19 anos, que apresentaram malformações anorretais ou doença de Hirschsprung, cujo tratamento cirúrgico tenha sido completado há pelo menos seis meses, que aceitaram participar da pesquisa. Foram excluídos os pacientes portadores de algum grau de comprometimento do desenvolvimento neuropsicomotor ou afecções neurológicas e urinárias que comprometessem o controle esfincteriano. Dos 71 casos, 31 eram de doença de Hirschsprung: forma clássica (aganglionose do reto-sigmoide), 23; aganglionose cólica total, sete; displasia neuronal intestinal, um. Do total, 22 eram do sexo masculino e nove do feminino. Os casos restantes, 40, eram de malformações anorretais: forma baixa, oito; intermediária e/ou alta, 15; persistência cloaca, quatro; fissura vésico-intestinal, um; atresia retal, um; não referida (crianças operadas em outros serviços encaminhadas à Instituição por motivo de obstipação intestinal), 11.

O grupo controle foi composto por 59 indivíduos hígidos, sem queixas de funcionamento intestinal e demograficamente semelhantes aos pacientes avaliados. Foram selecionados no ambulatório de cirurgia pediátrica e eram portadores de afecções cirúrgicas comuns, como hérnia inguinal e fimose. De forma semelhante aos grupos de estudo, os questionários foram enviados por correio.

As propriedades de medida estudadas foram a reprodutibilidade e a validade. Para avaliar a reprodutibilidade, o ICF e o QQVCFCA foram enviados duas vezes para os voluntários, por correio. Entre o primeiro e segundo envio, houve um intervalo de seis dias a 14 meses. Dos 71 pacientes iniciais, 22 não responderam aos segundos questionários enviados. Dos 49 pacientes restantes, 24 apresentam malformações anorretais e 25 apresentam doença de Hirschsprung.

Foi testada a validade construtiva e discriminativa. Para a avaliação da validade construtiva, os pacientes incontinentes responderam também ao questionário genérico conhecido como Short-Form 36, já validado em nosso meio. Os resultados obtidos foram correlacionados com os do QQVCFCA e, em seguida, compararam-se os resultados do ICF e do QQVCFCA. Foram ainda confrontados os resultados dos pacientes com malformações anorretais e com doença de Hirschsprung. A validade discriminativa foi avaliada com a aplicação dos questionários nos 71 pacientes operados e nos 59 indivíduos voluntários hígidos. Foram comparadas as pontuações médias de cada grupo no ICF, no QQVCFCA e em cada um de seus domínios.

Foram usados o índice de Bray-Curtis, o coeficiente de Pearson e o teste *t* de Student. O índice de significância estabelecido foi 0,05.

## Resultados

Com base no ICF, verificou-se que entre os 71 pacientes iniciais, 16 apresentaram má continência, 16 regular, 26 boa e 13 normal. Entre os 49 que participaram também da segunda etapa, verificou-se que dos 25 com doença de Hirschsprung, seis tiveram má continência, seis regular, nove boa e quatro normais. Também, entre os 24 com malformações anorretais, quatro tiveram má continência, cinco regular, 12 boa e três normais. Em suma, 45,07% (32/71) e 42,85% (21/49) dos pacientes apresentaram incontinência fecal nas duas etapas de aplicação do questionário.

Foi possível observar a reprodutibilidade dos resultados dos questionários ao longo do tempo por meio da aplicação deles nos mesmos pacientes em dois momentos distintos. O intervalo entre esses momentos variou de seis dias a 14 meses. A primeira aplicação foi chamada de ICF1 e QQVCFCA1 e a segunda de ICF2 e QQVCFCA2.

A similaridade entre os primeiros e segundos questionários provou-se satisfatória, segundo o índice de Bray-Curtis (>0,80). A reprodutibilidade ao longo do tempo manteve-se em todos os domínios avaliados [ICF, QQVCFCA, estilo de vida (EV), comportamento (COM), depressão (DEP) e constrangimento (CONS)], independentemente do intervalo entre a aplicação dos questionários (tabela 1).

**Tabela 1:** Comparação de similaridade entre ICF 1 e 2 e QQVCFCA 1 e 2 e seus domínios

Intervalo entre aplicação dos questionários	ICF1 × ICF2	QQVCFCA1 × QQVCFCA2	EV1 × EV2	COM1 × COM2	DEP1 × DEP2	CONS1 × CONS2
6 dias a 1 mês	0,95	0,96	0,94	0,97	0,93	0,92
2 meses a 3 meses	0,92	0,93	0,89	0,96	0,92	0,90
4 meses a 6 meses	0,92	0,96	0,94	0,97	0,95	0,94
6 meses a 14 meses	0,96	0,94	0,95	0,93	0,94	0,85
Todos	0,94	0,95	0,95	0,96	0,93	0,90

ICF, índice de continência fecal; QQVCFCA, questionário para avaliar a qualidade de vida relativa à continência fecal em crianças e adolescentes; EV, estilo de vida; COM, comportamento; DEP, depressão; e CONS, constrangimento. O número 1 após o critério se relaciona com a primeira aplicação dos questionários; o número 2, por sua vez, refere‐se à segunda aplicação dos mesmos questionários.OBS.: Índice de Bray‐Curtis: 0 a 0,19 – não similar; 0,20 a 0,39 – pequena similaridade; 0,40 a 0,59 – média similaridade; 0,60 a 0,79 – alta similaridade; 0,80 a 1,0 – máxima similaridade.

Para avaliar a validade construtiva, foi usada a correlação de Pearson e foram considerados valores 0,50 a 0,74 como correlação razoável e valores entre 0,75 e 0,99 como correlação forte. Observou-se correlação direta, com valor 0,57, entre os resultados do QQVCFCA e do SF-36, aplicados em 71 pacientes. Assim, temos que os resultados para qualidade de vida são similares com a aplicação do SF-36 ou do QQVCFCA. Comparando-se ICF e QQVCFCA, percebe-se a existência de uma dependência entre os mesmos (índice de 0,82). Há também uma forte relação entre o ICF com cada domínio do QQVCFCA individualmente (estilo de vida: 0,75; comportamento: 0,78; ausência de depressão: 0,78; ausência de constrangimento: 0,71). Dessa forma, quanto melhores os resultados de continência fecal, melhores a qualidade de vida e os parâmetros avaliados nos domínios.

Quanto à validade discriminativa, verificou-se que a qualidade de vida no portador de incontinência fecal está ainda comprometida globalmente, em todos os domínios e no ICF (médias de qualidade de vida: 11,2; estilo de vida: 3,1; comportamento: 3,0; depressão: 2,6; constrangimento: 2,3; e ICF: 10,4), quando comparado com os indivíduos voluntários hígidos (15,2; 3,9; 3,9; 3,6; 3,9; e 15,4; respectivamente), e há diferença estatística importante entre os grupos, ao aplicar-se o teste *t* de Student, com *p*<0,05 (tabela 2). Não houve ainda diferença significativa entre os resultados de crianças com malformações anorretais e com doença de Hirschsprung (tabela 3).

**Tabela 2:** Comparações dos ICF, QQVCFCA e seus domínios entre voluntários hígidos (grupo “normais”) e crianças com malformações anorretais ou doença de Hirschsprung (grupo “doentes”)

		Média	Desvio‐padrão	Percentil 25	Percentil 75	Mediana
*ICF*						
	Normais	15,37	1,0	15,0	16,0	16,0
	Doentes	10,4	4,48	6,0	15,0	11,0

*QQVCFCA*						
	Normais	15,2	0,9	15,0	15,7	15,5
	Doentes	11,2	3,3	8,6	14,8	11,2

*EV*						
	Normais	3,9	0,3	4,0	4,0	4,0
	Doentes	3,1	0,9	2,3	4,0	3,4

*COM*						
	Normais	3,9	0,2	3,8	4,0	4,0
	Doentes	3,0	0,8	2,5	3,8	3,1

*DEP*						
	Normais	3,6	0,31	3,4	3,8	3,6
	Doentes	2,7	0,83	2,1	3,5	2,7

*CONS*						
	Normais	3,9	0,2	4,0	4,0	4,0
	Doentes	2,3	1,11	1,3	3,6	2,0

MAR, malformações anorretais; DH, doença de Hirschsprung; ICF, índice de continência fecal; QQVCFCA, questionário para avaliar a qualidade de vida relativa à continência fecal em crianças e adolescentes; EV, estilo de vida; COM, comportamento; DEP, depressão; CONS, constrangimento. Feito teste *t* de Student, com *p* <0,05 e resultados estatisticamente diferentes.

**Tabela 3:** Comparações dos ICF, QQVCFCA e seus domínios entre crianças com malformações anorretais (MAR) e doença de Hirschsprung (DH)

		Média	Desvio‐padrão	Percentil 25	Percentil 75	Mediana
*ICF*						
	DH	10,0	4,4	5,5	13,5	11,0
	MAR	10,9	4,6	6,5	15,0	13,0

*QQVCFCA*						
	DH	11,2	3,4	8,1	15,0	11,0
	MAR	10,8	3,6	7,6	14,9	10,5

*EV*						
	DH	3,1	0,8	2,3	4,0	3,5
	MAR	3,1	0,8	2,3	4,0	3,0

*COM*						
	DH	3,1	0,8	2,5	4,0	3,3
	MAR	2,9	0,9	2,2	3,7	3,2

*DEP*						
	DH	2,7	0,8	1,8	3,4	2,5
	MAR	2,5	1,0	1,6	3,8	2,3

*CONS*						
	DH	2,2	1,2	1,2	3,6	1,6
	MAR	2,3	1,1	1,0	3,4	2,0

MAR, Malformações Anorretais; DH, Doença de Hirschsprung; ICF, Índice de Continência Fecal; QQVCFCA, Questionário para avaliar a qualidade de vida relativa à continência fecal em crianças e adolescentes; EV, Estilo de vida; COM, Comportamento; DEP, Depressão; CONS, Constrangimento.

## Discussão

A incontinência fecal pode ocorrer em algum grau de intensidade em cerca de 30% a 50% dos pacientes com malformações anorretais ou com doença de Hirschsprung,^6^ de modo semelhante ao que foi observado no presente estudo. Em geral atinge os âmbitos ocupacional, social, afetivo, esportivo, sexual e pode levar a transtornos psiquiátricos, com perda da independência individual. Está, portanto, intimamente relacionada com a QdV.^7^ Em artigo de revisão sobre diversos estudos, foi mostrado que pacientes com comprometimento do funcionamento intestinal têm também comprometimento da QdV, com essa correlação variando de pequena, média ou forte.^1^ Apesar do avanço nas técnicas cirúrgicas na cirurgia pediátrica, a incontinência fecal continua a ser um problema comum para os pacientes.^8^ Portanto, durante o atendimento ambulatorial, questionar apenas o número de evacuações e avaliar subjetivamente o status do paciente tem se mostrado insuficiente e superficial. É importante abranger o conceito de QdV, ou seja, o quanto esses problemas físicos/psíquicos/sociais afetam a funcionalidade e as expectativas do paciente. Por isso, a avaliação da mesma e da incontinência fecal deve ser feita por meio de métodos objetivos, quantitativos, reprodutíveis e que permitam seguimento longitudinal dos pacientes.

Na literatura, existem diversos instrumentos específicos e validados com tal objetivo, como o Fecal Incontinence Questionnaire,^9^ o The Fecal Incontinence Severity Index,^10^ o Fecal Incontinence Quality of Life (FIQL),^11^ o Hirschsprung's Disease Anorectal Malformation QoL Questionnaire (HAQL),^12^ o Medical Outcomes Study 36–Item Short–Form Health Survey^13^ e o Clinical Evaluation of Continence (Holschneider Criteria). Na presente investigação foram usados questionários traduzidos e adaptados para a língua portuguesa. Todos os casos foram avaliados pelo investigador principal (ALM) e foi considerada inexequível a participação dos autores originais por motivos de ordem prática. Tal conduta foi também ratificada pela Comissão de Ética e Pesquisa da Instituição.

O HAQL, por exemplo, foi elaborado especificamente para pacientes com malformações anorretais ou doença de Hirschsprung. É um questionário com 39-42 questões respondidas pelo próprio paciente ou por seus pais/responsáveis. Ele é composto por 10-11 subescalas, com itens relacionados a dieta laxante, dieta obstipante, diarreia, incontinência fecal, continência urinária, funcionamento social, funcionamento emocional, imagem corporal, sintomas físicos e funcionamento sexual. As respostas são fornecidas pelo paciente, pelos pais, ou por ambos, dependendo da idade. Com isso, existe um formulário para os pais das crianças de seis e sete anos; um para os pais juntamente com as crianças de oito a 11 anos e adolescentes de 12 a 16 anos; e uma forma adulta para os pacientes com 17 anos ou mais.^13^

O FIQL, por sua vez, validado em inglês em 2000, foi criado para avaliar a qualidade de vida em quaisquer pacientes com incontinência fecal. Ele é composto por 29 itens que avaliam quatro escalas: estilo de vida, comportamento, depressão e constrangimento. O mesmo questionário foi usado, independentemente da idade dos pacientes.^10^

Apesar de tantos instrumentos encontrados na literatura, nenhum se mostrou adequado para abordar a população-alvo do Instituto da Criança, São Paulo, Brasil. Parte da população atendida tem baixo nível cultural, o que dificulta/impossibilita a aplicação de questionários em outros idiomas. Há ainda questionários com opções consideradas complexas e de difícil entendimento, como o FIQL, com as seguintes opções: “concordo fortemente”, “concordo um pouco”, “discordo um pouco”, “discordo fortemente” e “não se aplica”. Ademais, parte dos pacientes deixa de frequentar regularmente o ambulatório cerca de 2-3 anos após o término do tratamento operatório, por má adesão ao tratamento ou mesmo por dificuldades de acesso ao sistema de saúde. Ainda, muitos pacientes residem em locais distantes e têm poucos recursos para deslocamento, o que dificulta a aplicação e feitura de entrevistas. Por fim, foram encontradas também questões consideradas inadequadas para crianças que envolvem sexualidade e situações de grave depressão. Desse modo, este estudo fez-se necessário e descreve a tradução, adaptação cultural e validação de dois questionários, o ICF e o QQVCFCA, congruentes com os pacientes com MAR ou DH atendidos. Foram usados como modelos o FIQL e o Holschneider Criteria, apresentados acima.

O processo de tradução, adaptação cultural e validação de questionários já tem sido feito em outros países. Minguez *et al*. fizeram a validação do FIQL para o espanhol, mantiveram as 29 questões e as aplicaram em pacientes com idade média de 60 anos com incontinência fecal de qualquer etiologia. Foi feita ainda a classificação de gravidade da incontinência e sua relação com os domínios do FIQL.^12^ Rullier *et al*. validaram o FIQL em francês, com um estudo semelhante ao de Minguez *et al*., e foram avaliados 100 pacientes com idade média de 57 anos.^7^ Na Suécia, Wigander *et al*. Fez a tradução e a adaptação cultural do HAQL. Os questionários tinham duas questões extras em relação ao HAQL original: a primeira perguntava se o paciente tivera dificuldade de entender as questões ou se as achara estranhas; a segunda, se existia alguma coisa que gostariam de acrescentar.^13^ Em nosso meio, Yusuf *et al*. validaram o FIQL para a língua portuguesa, porém sem adaptá-lo para crianças e adolescentes, a idade média dos pacientes foi de 52,8 anos.^14^

Em todos esses trabalhos, os questionários e instrumentos de avaliação de QdV e continência fecal sofreram um processo de tradução e adaptação cultural detalhado, para que se adaptassem a crenças, costumes, cotidiano, comportamentos e condições socioeconômicas da população-alvo. No presente estudo, a importância dessa etapa foi percebida por meio do número significativo de mudanças fundamentais nos questionários originais usados, tais como supressão de questões, modificação na redação de questões, criação de questões e alterações em opções. Além disso, foi necessária uma equipe multiprofissional que estruturou e, após uma avaliação inicial, modificou novamente os questionários. Esse processo exaustivo garante um aperfeiçoamento dos questionários finais.

Além disso, foram testadas a reprodutibilidade e a validade dos instrumentos para avaliar-se a confiabilidade. A reprodutibilidade provou-se estável, com índices sempre maiores do que 0,85. Dessa forma, conclui-se que houve uma similaridade importante nos resultados dos questionários em duas aplicações distintas, nos mesmos pacientes. O intervalo de tempo entre as aplicações variou de seis dias a 14 meses, sem que houvesse alterações nos resultados também. A reprodutibilidade provada no intervalo de tempo de seis dias a um mês é especialmente importante. Num período curto, é improvável que haja mudanças bruscas nas evoluções de todos pacientes, o que poderia comprometer os resultados. Num período maior, como o de 6-14 meses, a reprodutibilidade poderia não ter se comprovado devido a modificações no quadro clínico dos pacientes, se tivessem sido feitas intervenções com consequências mais marcantes.

Durante a fase de validação, observou-se uma correlação razoável entre o SF-36 e o QQVCFCA, com coeficiente de 0,57, considerado estatisticamente significativo. Isso confirma que o questionário analisado tem uma validade adequada quando comparado com um instrumento de sensibilidade já provada. Percebeu-se ainda uma relação direta entre o ICF e o QQVCFCA. Isso evidenciou que a qualidade de vida está afetada em pacientes com mau controle da continência fecal. Essa relação direta foi observada ainda entre o ICF e os domínios do QQVCFCA, de modo que quanto melhor a continência fecal, melhores os índices de estilo de vida, comportamento e ausência de depressão e de constrangimento. Por fim, na validação discriminativa, percebeu-se que os índices dos pacientes com malformações anorretais ou doença de Hirschsprung são inferiores aos da população controle, o que prova o impacto da continência fecal na qualidade de vida nos primeiros. Verificou-se que o maior impacto na qualidade de vida dos pacientes ocorre nos domínios de depressão e constrangimento.

Como limitações no estudo, pode-se citar a perda de 22 (30%) pacientes durante o processo de reprodutibilidade ao longo do tempo. Esses pacientes podem não ter entendido que se tratava de um processo de pesquisa, no qual o questionário seria enviado duas vezes intencionalmente e não acidentalmente. Assim, faz-se necessário melhor esclarecimento dos voluntários durante as atividades de pesquisa.

A associação, principalmente de malformações anorretais, com outras doenças congênitas e genéticas é comum e afeta uma porcentagem importante dos pacientes atendidos no ICr. Desse modo, pacientes com síndrome de Down, por exemplo, não foram contemplados nos critérios de inclusão, por apresentar outros fatores que pudessem interferir na avaliação de qualidade de vida, fatores de confusão. Assim, há uma grande necessidade da criação e validação de métodos específicos que permitam avaliar tais crianças, tanto em relação à continência fecal quanto em relação à qualidade de vida.

Por fim, é importante também que futuramente sejam estudadas as crianças mais novas, que não foram incluídas neste estudo por ser apenas em número de dez, consideradas não representativas do total. Nesse caso, pode-se pensar em diferentes questionários e formas de avaliação lúdicas, pela dificuldade de compreensão e abstração necessárias para as respostas.

Em conclusão, a criação do ICF e do QQVCFCA seguiu a tendência de diversos centros ao longo do mundo de criar e validar os próprios questionários. O processo de validação foi considerado satisfatório e forneceu instrumentos na língua portuguesa para avaliação de crianças com malformações anorretais e doença de Hirschsprung. Eles podem ser usados tanto na prática médica quanto na pesquisa. Constitui ainda um modelo que padroniza a avaliação, o que permite seu uso em outros centros.
